# Decompressive hemicraniectomy in pediatric malignant arterial ischemic stroke: a case-based review

**DOI:** 10.1007/s00381-023-06086-w

**Published:** 2023-07-26

**Authors:** Audrey Carlhan-Ledermann, Andrea Bartoli, Fabienne Gebistorf, Maurice Beghetti, Tornike Sologashvili, Monica Rebollo Polo, Joel Fluss

**Affiliations:** 1 Neonatology and Pediatric Intensive Care Unit, Department of Woman, Child and Adolescent Medicine, Geneva University Hospitals and Faculty of Medicine, University of Geneva, Geneva, Switzerland; 2 Neurosurgery Unit, Department of Clinical Neuroscience, Geneva University Hospitals and Faculty of Medicine, University of Geneva, Geneva, Switzerland; 3 Pediatric Cardiology Unit, Department of Woman, Child and Adolescent Medicine, Geneva University Hospitals and Faculty of Medicine, University of Geneva, Geneva, Switzerland; 4 Cardiovascular Surgery Unit, Department of Surgery, Geneva University Hospitals and Faculty of Medicine, University of Geneva, Geneva, Switzerland; 5 Pediatric Radiology Unit, Department of Radiology, Geneva University Hospitals and Faculty of Medicine, University of Geneva, Geneva, Switzerland; 6 Pediatric Neurology Unit, Department of Woman, Child and Adolescent Medicine, Geneva University Hospitals and Faculty of Medicine, University of Geneva, Geneva, Switzerland

**Keywords:** Pediatric stroke, Malignant edema, Craniectomy

## Abstract

**Purpose:**

Malignant stroke is a life-threatening emergency, with a high mortality rate (1–3). Despite strong evidence showing decreased morbidity and mortality in the adult population, decompressive hemicraniectomy (DCH) has been scarcely reported in the pediatric stroke population, and its indication remains controversial, while it could be a potential lifesaving option.

**Methods and results:**

We performed an extensive literature review on pediatric malignant arterial ischemic stroke (pmAIS) and selected 26 articles reporting 97 cases. Gathering the data together, a 67% mortality rate is observed without decompressive therapy, contrasting with a 95.4% survival rate with it. The median modified Rankin score (mRS) is 2.1 after surgery with a mean follow-up of 31.8 months. For the 33% of children who survived without surgery, the mRS is 3 at a mean follow-up of 19 months. As an illustrative case, we report on a 2-year-old girl who presented a cardioembolic right middle cerebral artery stroke with subsequent malignant edema and ongoing cerebral transtentorial herniation in the course of a severe myocarditis requiring ECMO support. A DCH was done 32 h after symptom onset. At the age of 5 years, she exhibits an mRS of 3.

**Conclusion:**

Pediatric stroke with malignant edema is a severe condition with high mortality rate if left untreated and often long-lasting consequences. DCH might minimize the vicious circle of cerebral swelling, increasing intracranial pressure and brain ischemia. Our literature review underscores DCH as an efficient therapeutic measure management of pmAIS even when performed after a significant delay; however, long-lasting morbidities remain high.

## Introduction

Arterial ischemic stroke (AIS) is defined as malignant or massive when the infarct area is associated with considerable brain swelling and mass effect. Such cerebral edema typically occurs after a large ischemic stroke and can rapidly lead to increased intracranial pressure and brain herniation, resulting in significant morbidity and mortality in affected adult patients [1, 3–6].

Compared to adults, children have a lower cerebral compliance and smaller subarachnoid and cisternal compartments, which may limit their capacity to tolerate cerebral edema and mass effect [4–7]. Indeed, younger age (after fontanelle closures) is paradoxically often cited as a risk factor due to the lack of cerebral atrophy that might play a “protective role” in old-age patients [1, 3]. Based on this assumption, and the known diagnostic delay for the recognition of childhood ischemic stroke [8, 9], one could expect that children could even be at higher risk for malignant arterial ischemic stroke (mAIS) than adults, but fortunately large hemispheric infarct occur less often in pediatric stroke. This is presumably due to a lower rate of large vessel occlusion related to distinct underlying causative factors [10].

The incidence of pediatric AIS is 2–7 in 100,000 children in developed countries, with a highest rate in children under 5 years and in boys [11, 12]. It is a serious condition with a 30-day mortality rate of 12.3% and long-term neurological deficit in more than 50% of survivors [1, 13]. In the adult population, malignant AIS (mAIS) most often involves the anterior circulation and occurs in approximately 20% of all AIS and is associated with a 80% of mortality rate [6].

The incidence of pediatric malignant AIS (pmAIS) is likely around 1%. Smith et al. reported an incidence rate of 1.3% of malignant middle cerebral artery infarct (MMCAI) in children [14], which is close to the 0.9% (34/3860) incidence of children with AIS who underwent a craniectomy found in the International Pediatric Stroke Study (IPSS) [15]. A higher incidence close to 12% was reported however in two other studies which include only anterior circulation stroke [16, 17]. In addition, Montgomery et al. suggested that up to 11% of posterior circulation ischemic stroke in children might result in malignant edema [7]. Finally, Andrade et al. found an incidence of MMCAI in children of 18%, but this high number was likely due to a referral bias [17, 18].

Initial therapeutic options in the setting of malignant stroke are limited to medical supportive measures aiming to maintain adequate homeostasis and to reduce, if possible, the developing brain swelling. Despite maximal supportive care, increasing intracranial pressure (ICP) and mass effect tend to frequently lead to transtentorial herniation that will precipitate a fatal outcome. Decompressive hemicraniectomy (DCH) is a surgical procedure that enables to acutely relieve intracranial pressure and to reduce the vicious circle of cerebral swelling, intracranial hypertension (IH), and ischemia. Despite strong evidence in favor of DCH in the adult population with stroke, its safety and efficacy in pediatric patients are still controversial, and current pediatric stroke guidelines do acknowledge this uncertainty [1, 4–6, 11, 19, 20].

Based on English-based literature review using 3 search engines (PubMed, Google Scholar, and Science Direct) completed by cross-references, we were able to identify 96 well-documented cases of pmAIS (Table 1). We collected data on gender, age, etiology (according to the CASCADE classification [21, 22]), clinical presentation, time to diagnosis, time to surgical procedure, type of intervention, surgical complication, long-term morbidity and mortality, and time of follow-up. Those data are discussed in detail in the next sections. A male predominance with a sex ratio of 3 male for 1 female and a median age of 9.3 year was found.

**Table 1:** Alphabetical list according to the 1st author of reviewed publications; summary of study design, and main findings

Date	Study	Sex	Age (month)	ICP sign ^1^anisocoria ^2^deterioration of consciousness ^3^other	Seizure	Vascular territory	CASCADE classification^6,7^ ^O: other or undetermied etiology^ ^A: artheriopathy^ ^CE: cardioembolic^	Time from AIS to MAIS (hours)	Time from MAIS to imagery (hours)	Treatment delay (hours) ^1^initial symptoms to DCH ^2^malignant symptoms to DCH	Treatment	Surgical complication	Outcome (mRS)	Follow-up (month)
2009	Aghakhani et al. [23]	M	132	1, 2	No	MCA	O	NA	NA	2^1^, NA^2^	R ST DCH	None	3	96
2016	Andrade et al. [18]	M	169	NA, 2	No	MCA	*41.7% CE, 33.3% A, 25% O	28^*^	NA	96^1^	DCH	NA	1	19*
		F	63	NA, 2	Yes > 5 min	MCA	*	28^*^	NA	24^1^	DCH	NA	2	19*
		M	156	NA, 2	Yes > 5 min	MCA	*	28^*^	NA	/	No	NA	2	19*
		M	204	NA, 2	No	MCA	*	28^*^	NA	/	No	NA	4	19*
		M	74	NA, 2	Yes > 5 min	MCA	*	28^*^	NA	/	No	NA	3	19*
		M	108	NA, 2	No	MCA	*	28^*^	NA	120^1^	DCH	NA	4	19*
		M	169	NA, 2	Yes > 5 min	MCA	*	28^*^	NA	48^1^	DCH	NA	4	19*
		M	54	NA, 2	No	MCA	*	28^*^	NA	24^1^	DCH	NA	5	19*
		M	36	NA, 2	Yes > 5 min	MCA	*	28^*^	NA	48^1^	DCH	NA	5	19*
		F	168	NA, 2	Yes > 5 min	MCA	*	28^*^	NA	/	No	NA	Death	/
		M	180	NA, 2	Yes > 5 min	MCA	*	28^*^	NA	/	No	NA	Death	/
		F	120	NA, 2	Yes > 5 min	MCA + ACA	*	28^*^	NA	12^1^	DCH	NA	Death	/
1981	Bergen et al. [24]	M	114	2, 3	No	VA	A	168	2	174^1^, 6^2^	VD	NA	0	30
2018	Bigi et al. [16]	NA	163	NA	NA	ICA	O	NA	3	NA^1,2^	MT/IAT, ST DCH	NA	1	48
		NA	182	NA	NA	MCA	O	NA	6	NA^1,2^	IAT, ST DCH	NA	4	48
		NA	169	NA	NA	ICA	O	NA	3	NA^1,2^	MT / IAT, ST DCH	NA	3	48
		NA	105	NA	NA	MCA + ACA	O	NA	4	NA^1,2^	IAT, ST DCH	NA	3	6
		NA	176	NA	NA	BA	CE	NA	4	NA^1,2^	MT, ST DCH	NA	NA	NA
1987	Brawn et al. [25]	M	60	2	Yes	Ct	CE	12	NA	NA^1,2^	VD	NA	1	24
1997	Carter et al. [26]	M	132	1, 2	No	MCA + PCA	A	NA	NA	24^1^, NA^2^	ST DCH	NA	1	12
2005	Curry et al. [27]	NA	192	NA	NA	NA	NA	NA	NA	NA^1,2^	DC	NA	1	12
		NA	216	NA	NA	NA	NA	NA	NA	NA^1,2^	ST DCH	NA	1	12
2009	Farooq et al. [8]	F	19	2	No	MCA + ACA	CE	40	36	42^1^, 38^2^	ST DCH	None	1	6
1972	Fischer et al. [28]	M	5	2, 3	Yes	Ct	O	NA	NA	NA^1,2^	IT DCH	NA	1	NA
1982	Harbaugh et al. [29]	M	156	2	No	Ct	A	120	NA	NA^1,2^	VD	NA	1	3
2007	Kirton and deVeber [30]	M	132	1, 2	No	MCA + PCA	CE	NA	0,75	48^1^, 6^2^	ST DCH	None	3	6
2016	Lammy et al. [31]	M	192	2	No	MCA	O	NA	12	12^1^, NA^2^	ST DCH	None	1	3
2003	Lee et al. [32]	M	72	1, 2	No	MCA	CE	24	2	2^1^, NA^2^	ST DCH	None	2	21
2012	Lee et al. [33]	M	33	1, 2	No	MCA	O	NA	NA	19^1^, NA^2^	ST DCH	None	1	36
		F	17	1, 2	No	MCA	CE	NA	NA	NA^1^, 5^2^	ST DCH	None	1	41
		M	26	1, 2	No	MCA	O	NA	NA	18^1^, NA^2^	ST DCH	None	3	68
		M	17	1, 2	No	MCA	O	NA	NA	14^1^, NA^2^	ST DCH	None	1	38
2019	Lehman et al. [34]													
	*N* = 29	59% F	73	1: NA 2: 76%	24%	100% anterior 24% anterior + posterior	34% A, 45% CE	NA	NA	48% 24 ^1^, NA^2^	DCH	NA	59% 2, 10% death, 31% NA	NA
	*N* = 5	20% F	124	1: NA 2: 80%	20%	80% posterior 20% anterior + posterior	20% A, 60% CE	NA	NA	100% 48^1^, NA^2^	DCH	NA	60% 0.5, 40% NA	NA
2002	Leonhardt et al. [35]	F	180	NA	NA	MCA	A	NA	NA	12^1^, NA^2^	ST DCH	None	2	24
1994	Miyata et al. [36]	F	132	2	No	Ct	A	192	2	NA^1,2^	IT DCH	None	1	72
1973	Momose and Lehrich [37]	M	5	2, 3	Yes	Ct	NA	NA	NA	NA^1,2^	IT DCH	NA	1	NA
2012	Montgomery et al. [7]	M	108	2, 3	No	PICA + SCA + AICA	O	48	24	72^1^, 24^2^	ST DCH	NA	1	24
		F	72	2, 3	No	PICA + PCA + AICA + SCA	A	72	24	72^1^, 24^2^	IT DCH	Hemorrhage	3	96
		M	120	2, 3	No	AICA + SCA + PCA	A	48	48	72^1^, 24^2^	IT DCH	NA	1	48
		M	84	1, 2, 3	No	PCA	A	24	2	24^1^, 2^2^	IAT, IT DCH	NA	2	60
1987	Perez-Higuera et al. [38]	M	108	2, 3	No	Ct	A	NA	NA	NA^1,2^	IT DCH	None	3	6
2013	Rahme et al. [17]	F	204	NA, 2	NA	MCA	A	NA	NA	/	No	/	Death	/
		M	84	NA, 2	NA	MCA	O	72	NA	NA^1,2^	ST DCH	NA	3	1
2008	Ramaswamy et al. [2]	M	48	1, 2, 3	Yes	MCA + ACA	A	24	2	32^1^, 8^2^	ST DCH	NA	2	72
		F	156	1, 2, 3	No	ACA + MCA + ICA	A	48	2	60^1^, 12^2^	ST DCH	NA	1	18
		M	128	1, 2, 3	Yes	MCA + MCA	CE	48	2	NA^1^, 8^2^	ST DCH	NA	3	12
		M	26	1, 2	Yes	MCA	CE	24	2	35^1^, 8^2^	ST DCH	NA	2	6
2013	Shah et al. [1]	M	264	1, 2, 3	No	MCA	O	68	2	69^1^, 2^3^	ST DCH	Cellulits	3	36
		M	168	1, 2, 3	No	MCA + ACA	O	24	6	30^1^, 6^2^	ST DCH	None	3	24
		M	180	1, 2	Yes	MCA	A	72	2	72^1^, 2^2^	MT, ST DCH	Bone flap infection	2	36
2011	Smith et al. [14]	M	142	NA, 2	NA	MCA	A	NA	NA	144^1^, NA^2^	Bilateral ST DCH	NA	2	86
		M	21	NA, 2	NA	MCA + ICA	A	NA	NA	27^1^, NA^2^	R ST DCH	NA	3	64
		F	32	NA, 2	NA	MCA + ICA + ACA	CE	NA	NA	60^1^, NA^2^	L ST DCH	NA	3	31
		M	78	NA, 2	NA	MCA + PCA	A	NA	NA	24^1^, NA^2^	R ST DCH	NA	2	80
		M	106	NA, 2	NA	MCA + PCA	A	NA	NA	291^1^, NA^2^	L ST DCH	NA	3	66
		M	168	NA, 2	NA	MCA	CE	NA	NA	48^1^, NA^2^	R ST DCH	NA	4	49
		M	28	NA, 2	NA	MCA	A	NA	NA	23^1^, NA^2^	L ST DCH	NA	3	20
		F	156	NA, 2	NA	MCA	A	NA	NA	/	No/ICPM	/	Death	/
		M	157	NA, 2	NA	MCA	CE	NA	NA	/	No/ICPM	/	Death	/
		M	128	NA, 2	NA	MCA	A	NA	NA	/	No/ICPM	/	Death	/
2006	Tan et al. [39]	F	29	1, 2, 3	No	MCA	CE	24	2	NA^1^, 2^2^	L ST DCH	NA	4	5
2016	Yamaguchi et al. [40]	F	72	1, 2, 3	Yes	MCA	A	96	12	116^1^, 20^2^	ST DCH	None	4	4
2019	Our case	F	24	1, 2	No	MCA	CE	110	30	112^1^, 32^2^	R ST DCH	Parenchymal hernia	3	36
Median N = 97		63%M/37% F	101	100% 1, 2 or 3; 61% 1, 91% 2	31% yes	See Table 2	CE 36%, A 39%, O 18%	41	9, 4	48, 2^1^ 12, 7^2^	91% surgery	22%	11% mortality (67% without DCH, 4.5% with DCH); mRS 2.1 with DCH, 3 without DCH	31.8

*ACA* anterior cerebral artery, *AICA* anterior inferior cerebellar artery, *ASD* atrial septal defect, *AVM* arteriovenous malformation, *BA* basilar artery, *Ct* cerebellar territory (not specified), *ECMO* extracorporeal membrane oxygenation, *IAT* intra-arterial thrombolysis, *ICA* internal carotid artery, *ICP* increasing intracranial pressure, *IT* infratentorial, *L* left, *MCA* middle cerebral artery, *MT* mechanical thrombectomy, *PCA* posterior cerebral artery, *PICA* posterior inferior cerebellar artery, *R* right, *SCA* superior cerebellar artery, *ST* supratentorial, *VA* vertebral artery, *NA* not available, *VD* ventricular drainage

*Median of the 12 cases by Andrade et al.

## Historical background

The recognition that stroke could occur in infants and children has veritably emerged in the past three decades, despite the fact that the first stroke in a child was reported by T. Willis back in 1667 [23, 41] and that Freud himself pointed to the vascular origin of most congenital hemiplegia. Much efforts have been first devoted to elucidate etiological mechanisms of childhood arterial ischemic stroke that can be grossly divided in two main categories: those with a vessel wall abnormality, i.e., arteriopathy, and those of cardioembolic origin [11, 30, 42–44]. While a vast majority of children with arteriopathy (apart from children with preexisting genetic condition) are previously healthy, most cardioembolic stroke occur in children with congenital cardiac malformation rather than acquired cardiac disorders.

The emergence of pediatric stroke specialists and dedicated centers along with better imaging has contributed to an increased awareness regarding neonatal and pediatric AIS that in turn has shortened the diagnostic delay to a time frame that now enables to consider, in selected pediatric patients, hyperacute therapies similar to adult protocols [11, 12, 45–47]. Albeit rare in the pediatric population, the occurrence of pmAIS is well reported and almost always dramatic, but there is still a paucity of data in the literature regarding its optimal management and the role of DCH [11, 15].

The earliest technique of opening the human skull, named trephination, can be traced to at least 12,000 years before Christ. The surgical procedure with pathophysiological concepts and surgical techniques resembling our modern DCH was published in 1901 by Kocher [48], who considered that pressure relief by surgical trepanation was indicated in all cases of intracranial hypertension. In 1908, Harvey Cushing described the technic of subtemporal decompressive craniectomy (DC) for traumatic brain injury [49]. Decompressive craniectomy following malignant stroke began to emerge in the 1950s, with a first reported case by Arthur King in 1951 [6, 50].

The available evidence about DC comes from multiple randomized trials in adult population with severe traumatic brain injury (DECRA and RESCUEicp) as well as for malignant middle cerebral artery infarct (DECIMAL, DECIMAL II, DESTINY, DESTINYII, HAMLET) and can be summarized as follows:

Unilateral or bifrontal DC used as a last tier therapy for severe, sustained, and refractory post-traumatic intracranial hypertension leads to a substantial mortality reduction but increased rate of severe disabilities [51, 52]. In contrast, early neuroprotective bifrontal DC for mild to moderate intracranial hypertension has not been shown to be superior to medical management for adult patients with diffuse traumatic brain injury [53].

Concerning malignant stroke, there is substantial evidence that DC is associated with both reduced mortality and improved rate of moderate to good survival outcome if performed in adults up to 60 years and when performed within 48 h after stroke onset [54].

In both conditions, surgical decompression, while increasing survival, still remains associated with long-lasting disabilities that raise important ethical issues [55, 56].

In the pediatric population, DCH is widely accepted for elevated ICP secondary to traumatic brain injury [57] and has also been reported after infectious encephalitis, subarachnoid hemorrhage, hemorrhagic stroke, and cerebral sinus venous thrombosis [1]. The first pediatric case of DCH in the setting of pmAIS was published in 1972 [28, 28]. Less than hundred cases have been reported since then (Table 1).

## Clinical presentation and presumed etiology of pediatric malignant arterial ischemic stroke

The most common symptoms of childhood ischemic stroke include hemiparesis and hemifacial weakness (67–90%), speech or language disturbance (20–50%), vision disturbance (10–15%), ataxia (8–10%), headache (20–50%), and altered mental status (17–38%) [11, 58]. The latter three manifestations are typically seen in posterior circulation stroke [59]. Seizures at stroke onset occur in around 20% of cases, mostly in young children [58, 60].

Based on retrospective study of Andrade et al., pmAIS is strongly associated with older age, prolonged seizures during the first 24 h (odds ratio 25.51, *p* = 0.005), and higher initial PedsNIHSS score (odds ratio 1.22, *p* = 0.006). In their review, all children with the combined presence of age ≥ 2 years, seizures lasting ≥ 5 min, and an initial PedsNIHSS score ≥ 8 points developed a malignant stroke [18]. In the retrospective study of Lehman based on data from the IPSS, seizures occurred in a quarter of children with anterior circulation stroke who underwent a craniectomy [15]. In our review, we found that all children with pmAIS exhibit in the course of their illness raised ICP symptoms. Occurrence of a unilateral mydriasis (61% (19/31)) and secondary deterioration of the level of consciousness (91% (81/89) are well-identified features. Those signs along with seizures (present in 31% (24/77) of the subjects) should promptly raise the clinical suspicion of pmAIS [61].

The etiology of stroke in our studied population is diverse. According to the CASCADE classification, the etiology was secondary to an arteriopathy in 38% (36/94), cardioembolic in 36% (34/94), and unknown (or other) in 18%. A small number (7/97) of pmAIS occurred after intra-arterial thrombolysis (IAT) or mechanical thrombectomy (MT) raising the concern that those interventions could per se be a risk factor for malignant stroke. Bigi et al. were able to show that patients with pmAIS compared to non-malignant AIS received more frequently recanalization treatment than standard care, but pmAIS children had higher baseline pedNIHSS score at presentation than patients with AIS. They concluded that the higher frequency of pmAIS in the recanalization group was essentially reflecting more severe brain infarcts rather than a complication attributable to the treatment modality itself. Unfortunately, the time interval from recanalization treatment to malignant manifestations was not reported [16].

The topography of pmAIS is shown in Table 2. In 26% of cases (16/61), more than one infarcted territory was present (Table 1).

**Table 2:** Percentage of artery location of stroke

**Artery location**	**Percentage**
Middle cerebral artery MCA	77% (47/61)
Posterior cerebral artery PCA	11.5% (7/61)
ACA	11.5% (7/61)
ICA	6.5% (4/61)
VA	1.5% (1/61)
BA	1.5% (1/61)
Cerebellar territory 1. Cerebellar territory (not specified) 2. PICA 3. SCA 4. AICA	15% (9/61) 10% (6/61) 3% (2/61) 5% (3/61) 5% (3/61)

## Diagnosis

Definite diagnosis of malignant stroke is made after clinical suspicion by brain CT scan or magnetic resonance imaging (MRI) with the visualization of an acute infarct area with restricted diffusion on DWI and mass effect [1, 3–6, 62].

As already pointed out, diagnosis delay of pediatric stroke remains unfortunately frequent [9, 47, 58, 62]. The major causes of delays include delayed consideration of stroke among frontline heath providers, number of medical conditions that mimic stroke in children, lack of pediatric stroke guidelines, and delays in accessing MRI, often related to the need for sedation or anesthesia [63, 64]. Such delays can also impact therapies like DCH. In cases of AIS after cardiac surgery, stroke diagnosis is unfortunately often made when imaging is obtained for other reasons (cardiac arrest, extracorporeal membrane oxygenation cannulation) [11]. In our in-depth analysis of the 97 pmAIS cases, the precise timing of events was often lacking. Therefore, extrapolation of timing was applied in order to get a crude estimate of the various delays.^1^ The median time from symptoms onset and malignant manifestations was 41 h (range 12–192 h) with information available in 33/97 cases. The diagnosis delay from malignant presentation to imaging was 9.4 h (range 0.75–48 h) with sufficient information provided in 25/97 cases. The median treatment delay since initial manifestations was 48.2 h and since malignant symptoms was 12.7 h (range 2–38 h) with, respectively, available information in 56/88 and 18/88 cases.

## Management, prognosis, and outcomes

In our pooled analysis, clinical deterioration occur within 41 h after stroke onset highlighting the importance of close clinical and radiological monitoring [7, 12, 45, 47, 59].

The value of ICP monitoring is missing in large AIS. Some authors suggest that ICP monitoring can paradoxically delay surgery; indeed, in a small case series, 3 children with ICP monitoring died before surgical management was considered after the rise of ICP [14].

The surgical aim, through a fronto-temporo-parietal hemicraniectomy, is decompression of the corresponding swollen and infarcted area, in order to prevent escalating brain edema, which in turn can cause reduced brain perfusion and further worsening. Large bone flaps are recommended. If this “vicious circle” is not interrupted, brain herniation with subsequent brainstem compression and fatal outcome will irremediably occur [55]. In case of cerebellar infarction, an infratentorial DC is performed [36].

Andrade et al. shows that survivors of malignant middle cerebral arterial infarct (MMCAI) had significantly more residual neurological deficits compared with children without it, but nearly all were ambulatory and speech was preserved in most (median Pediatric Stroke Outcome Measure of survival 3.2/10 (range 0.5–9))[18]. Lehman et al. reviewed 34 cases and shows that the outcome is better after posterior than anterior circulation stroke [15]. Shah et al. described 3 new cases and reviewed 26 cases of the literature (including Smith’s cases) of DCH following pmAIS [1, 14]. The data suggest that a good outcome is possible even in front of brain herniation, low preoperative GCS score, involvement of multiple vascular territories, or longer time to surgery (mean of 43 h, range of 2–291 h). All children survived with good to moderate outcome [1]. Beez et al. reviewed 28 pmAIS cases described in the literature (including Smith’s and Shah’s cases). They show that 84% of children had preoperative anisocoria indicating herniation. Nevertheless, their outcome appears to be better than in adults, with 96% of cases showing a fairly good outcome [6]. Based on literature of transtentorial herniation after traumatic brain injury in adults and children, bilaterally absent pupillary function and low initial GCS are associated with poor prognosis, while anisocoria remains associated with a good outcome or a moderate disability [50, 65]. A systematic review in children (*n* = 172) who underwent DC after increased ICP shows that patients without signs of cerebral herniation had a better outcome than patient with unilateral or bilateral mydriasis (73% vs 60% vs 45%, respectively) [66].

We measure the outcome using the modified Rankin score [67]. For case reports without mention of the mRS, we estimated an mRS based on available clinical data or other outcome scales such as the PSOM [68] with a possible risk of bias. The median modified Rankin score (mRS) as a proxy for morbidity outcome was 2.1 after surgery. For the remaining 33% who survived without surgery, the mRS was 3. Among the survivors, 2% (1/55) had no deficit, 51% (28/55) had only mild deficit (mRS 1–2), 40% (22/55) had moderate deficits (mRS 3–4), and 4% (2/55) had severe deficit (mRS 5–6). The median time of follow-up was 31.8 months (range 1–96 months).

In adults, a 50% reduction in mortality rate after DCH in mAIS [6] was observed. According to Shah et al., malignant stroke has a mortality rate > 50% [1]. In the retrospective study by Andrade et al., 25% of children with MMCAI died compared with less than 4% in the non-MMCAI group [18]. In our pooled analysis, the majority (91%) of reported cases underwent a surgical procedure. Without DCH, the mortality rate was 67%, compared to 4.4% with DCH. All DCH were performed after the onset of IH signs, including 61% with evidence of anisocoria. Those numbers illustrate the persisting benefit of DCH even after brain herniation. In the largest series in pediatric population, 95% of children who had DCH survived [15]. In most relevant publications, survival rate was close to 90–100% after DCH [1, 6, 14, 18].

Ferriero et al. recommend to perform early prophylactic DCH in children with large volume infarct within the first 24 h or to monitor for potential swelling during the first 72 h [11]. Grant recommends considering DCH in patient showing early signs of neurological deterioration, herniation, or refractory increased ICP [69]. Smith et al. also recommend to consider DCH in children with a large AIS with deterioration of the level of consciousness or GCS score of 7 or less [14].

Complications of DCH are insufficient decompression, infection, hemorrhage, contralateral subdural effusions, external cerebral herniation, leakage of cerebrospinal fluid, sinking flap syndrome, delayed hydrocephalus, and subdural hematomas [1]. DCH in children can lead to significant intraoperative blood loss of up to 50% of estimated blood volume and thus requires adequate preparation [6]. In our analyses, 22% (4/18 cases mentioned) had complications after DCH. One case had acute hemorrhage and right cerebellar edema motivating bilateral DCH, one case had cellulitis, one case had bone flap infection, and our case had parenchymal hernia.

## Exemplary case description

**Fig. 1:**
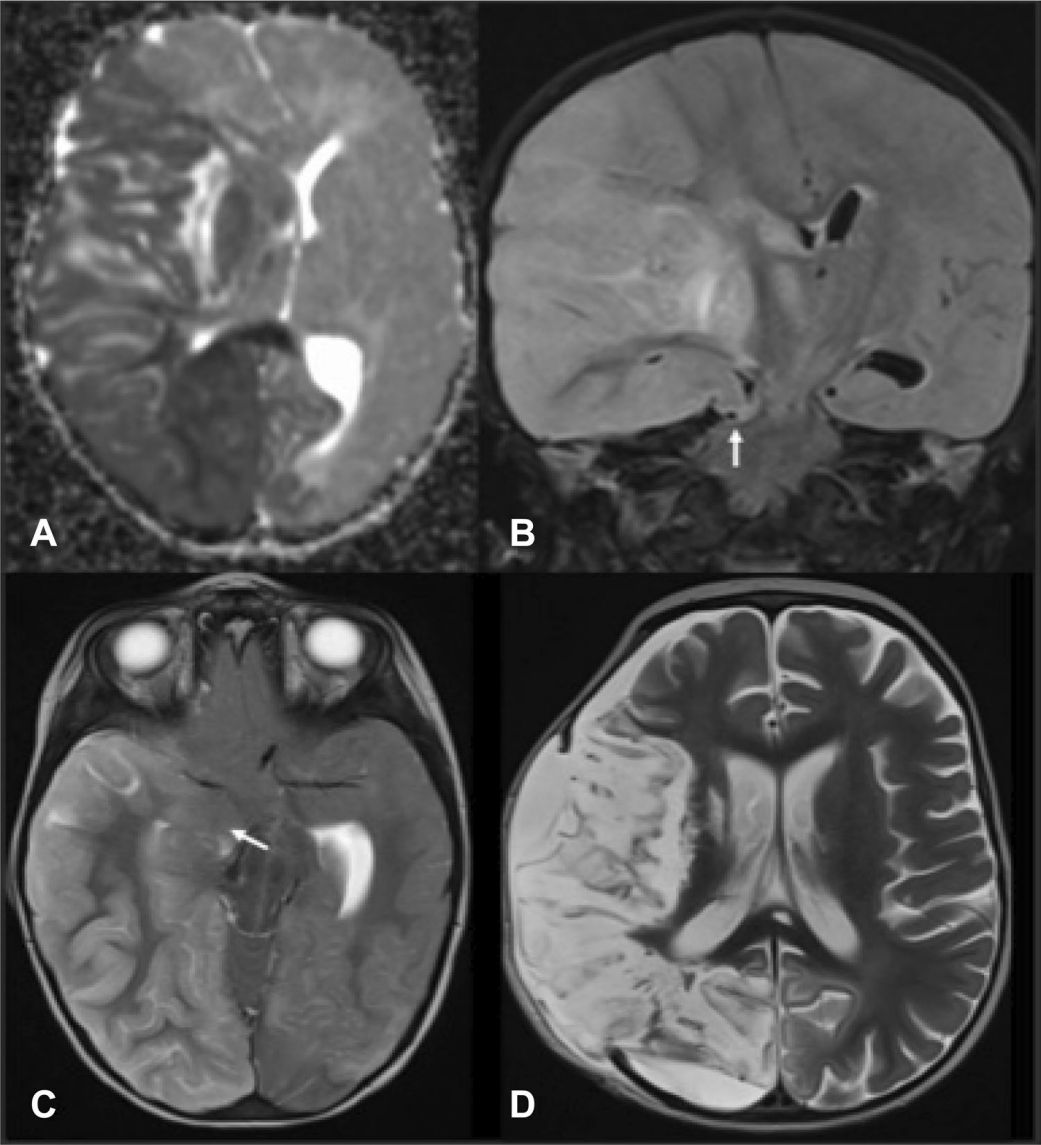
Anisotropy diffusion coefficient (ADC) map. Initial MRI shows a large subacute ischemic lesion on the right MCA and PCA territory. Restricted diffusion of the splenium and contralateral occipital mesial cortex involvement (**A**). Coronal T2 FLAIR and axial T2. Deviation of the midline with transtentorial herniation of the frontobasal parenchyma, signs of uncal herniation with effacement of the interpeduncular cistern, and severe mass effect on the midbrain (*arrows*) are also evident (**B**, **C)**. Follow-up MRI performed 4 weeks later shows an extensive cortico-subcortical volume loss of the affected parenchyma, including the right putamen. Focal lesions are depicted in the right thalamus and splenium. A minimal parenchymal herniation through the flap is also noted (**D)**

We report the case of a previously healthy 2-year-old girl, admitted to the pediatric intensive care unit (PICU) within the setting of severe enterovirus myocarditis, complicated by major cardiac dysfunction, multiple episodes of arrhythmia, and cardiac arrests who underwent extracorporeal membrane oxygenation (ECMO) with therapeutic anticoagulation. While on ECMO, a left hemisyndrome was progressively observed and subsequently a right eye anisocoria noticed. A possible cerebrovascular event was considered, but imaging had to be postponed after decannulation and removal of pacing wires. Brain MRI (Fig. 1) performed 110 h after initial symptom onset (according to chart review) and about 30 h after the first observation of the anisocoria demonstrated a massive right middle cerebral artery stroke with malignant edema and ongoing cerebral transtentorial herniation.

Despite the delay in diagnosis, and after family consent, it was decided to perform an urgent (about 2 h after MRI) right supratentorial decompressive craniectomy. Simultaneously, a large thrombus in the left ventricle and aortic root was identified, requiring anticoagulation by low-molecular weight heparin introduced 5 days after DCH for 3 months followed by preventive low-dose aspirine and a surgical thrombectomy after stabilization of the neurological situation.

DCH was complicated by parenchymal hernia through the head tip. The autologous flap was replaced 1 month after DCH. Three months later, the autologous flap showed signs of bony resorption, requiring a cranioplasty with an heterologous flap.

Follow-up neuroimaging at 1 month demonstrated a right hemispheric global atrophy (Fig. 1D). Clinically, she rapidly exhibited left spastic hemiplegia with little residual function, left visual field hemianopsia, and developed with a focal epilepsy, initially drug resistant. At 36-month follow-up, significant improvement was fortunately seen. The child was able to walk independently, to use her right arm as support, and to produce short sentences corresponding to a mRS of 3. Epilepsy was in full remission, and antiepileptic drug was progressively withdrawn. Full cognitive assessment shows mild global developmental delay, and a specialized school was considered.

## Conclusion

Pediatric malignant stroke remains a dramatic event with significant mortality and morbidity. Because of the rarity of the entity, a prospective multi-institutional study to determine optimal management is difficult to set up, and data from small series and retrospective reviews need to be cautiously evaluated.

In our illustrative case, DCH was performed urgently soon after a definitive diagnosis of mAIS was achieved, but the earlier clinical manifestations were unfortunately overlooked. Our case is noteworthy because the onset of stroke occurred in a sick child under ECMO which is a well-known pitfall [70, 71]. This case illustrates therefore the crucial need for improving recognition of AIS in all settings, including in-hospital units.

The literature shows that the best factors to differentiate PAIS from pmAIS are high PedsNIHSS score at onset, prolonged seizures, and anisocoria [18, 61]. This finding emphasizes the importance of implementing the PedNIHSS scale as a practical bedside tool in childhood stroke.

Thrombolysis and/or endovascular thrombectomy are possible risk factors for the occurrence of pmAIS, but current data do not allow to draw firm conclusions and are still weak due to recent implementation of those techniques in pediatric stroke. An intensive surveillance in dedicated units is clearly mandatory after recanalization therapy in children and adolescents with large arterial stroke.

This case, along with our literature review, highlights the fact that DCH should be considered as a potentially lifesaving therapy in pmAIS, even if performed late and/or in the setting of brain herniation signs. Even if the mRS is better with early DCH, we still recommend performing a DCH in case of delayed diagnosis. Any change in the level of consciousness, moreover in the event of prolonged seizures, should promptly raise the suspicion of mAIS and the consideration of decompressive craniectomy, but expected compromised outcome should be discussed with the family according to imaging findings. Although better outcome after delayed DCH in pediatric population can be anticipated compared to adult, one should not underestimate significant long-term morbidity. One should also acknowledge the difficulty to properly measure and interpret outcome based solely on the mRS [72].

The potential benefit of hemicraniectomy in large pediatric stroke confirms the importance of a multidisciplinary expertise within a tertiary center. Excessive reliance on ICP monitoring and values can prove counterproductive and might delay management until irreversible herniation has occurred. Newly available bedside neuromonitoring including transcranial Doppler, pupillometry, and quantitative EEG are promising tools to detect early deterioration [73, 74].

The implementation and timely activation of pediatric stroke protocols are critical factors in order to improve acute phase management and surveillance. Special attention should be given to large infarcts and an early DCH considered.

## Data Availability

All data generated or analyzed during this study are included in this published article.

## Abbreviations

ACA: Anterior cerebral artery
AICA: Anterior inferior cerebellar artery
AIS: Arterial ischemic stroke
BA: Basilar artery
Ct: Cerebellar territory (not specified)
DC: Decompressive craniectomy
DCH: Decompressive hemicraniectomy
IAT: Intra-arterial thrombolysis
ICA: Internal carotid artery
ICP: Intracranial pressure
IH: Intracranial hypertension
mAIS: Malignant arterial ischemic stroke
MCA: Middle cerebral artery
MMCAI: Malignant middle cerebral artery infarct
MRI: Magnetic resonance imaging
mRS: Modified Rankin score
MT: Mechanical thrombectomy
PAIS: Pediatric arterial ischemic stroke
PCA: Posterior cerebral artery
PICA: Posterior inferior cerebellar artery
pmAIS: Pediatric malignant arterial ischemic stroke
SCA: Superior cerebellar artery
VA: Vertebral artery

## Footnotes

1 (i.e., urgently or immediate would be 2 h, few hours 12 h less than 2 days would be 36 h, and few days would be 72 h).
